# Tincture Cin Honœ Ferrata

**Published:** 1853-09

**Authors:** Samuel Sims


From the American Journal of Pharmacy.

*Tinctura Cin honæ Ferrata.*—By SAMUEL SIMS.

Huxham's Tincture, the officinal Compound Tincture of Peruvian Bark, cannot be combined with any of the ordinary chalybeates without an offensive decomposition, seriously affecting its apparent, if not its real characteristics. These reactions were examined at the solicitation of Dr. J. F. Meigs, and a tincture prepared, so modified that it is no longer incompatible with the salts of iron. Sixteen grains of Ammonio Citrate of Iron are dissolved in each fluid ounce of this modified tincture, together constituting what has been denominated, for distinction, "Ferrated Tincture of Bark."

It is readily prepared by digesting in the Edinburgh Compound Tincture of Cinchona, sufficient hydrated sesquioxide of iron to completely eliminate the cincho-tannin, whether pure, oxidized, or combined. One ounce of hydrated sesquioxide, dried at a temperature not exceeding 130° Fahr., usually suffice for one gallon of the tincture. After the filtration, the tannate and excess of oxide should be washed with boiling alcohol to remove any trace of alkaloid which may have been in combination with the tannin and precipitated with it. This alcoholic solution may be evaporated to dryness, the product dissolved in a little water acidulated with citric acid, and added to to the filtrate along with the proper quantity of iron salt. It differs little in appearance from the ordinary Huxham's tincture, exceedingly agreeable, and in teaspoonful doses has become a very energetic invigorative, admirably adapted for administration in those cases of weak and languid habits of children and females, where the body is in a pallid or flaccid state, and very susceptible of fatigue or morbid action.

The ferrated tincture is not solely dependent on the quinia and iron it contains for its value as a curative agent. The grateful, and by no means inefficient adjuvants, the orange peel and snake root, and the other proximate principles of cinchona, independent of quinia, are by no means to be overlooked, and cannot be replaced by salts of quinia and iron alone, however scientific their artificial combinations may appear.

Fine French brandy employed as the menstruum, yields a still more grateful preparation, and should be substituted in all cases for the diluted alcohol, when the additional expense is no obstacle.

A quantitative examination of the cinchona alkaloids contained in this tincture, both before and after the elimination of the tannin, was made without appreciable difference in the result. A portion of the tincture as I have prepared it having been delivered to Prof. Booth to determine its iron constituents with accuracy, elicited the following statement:

PHILADELPHIA, Aug. 3d, 1853.

DEAR SIR:—The Ferrated Tincture of Bark, which you submitted to me for analysis, yields 4.23 grains of sesquioxide of iron to the fluid ounce.

Respectfully yours,

JAS. C. BOOTH.

MR. SAMUEL SIMS, Philadelphia.

The sesquioxide in 100 grains of the ammonio-citrate of iron was accurately determined to be 25.84 grains, consequently 4.23 grains of sesquioxide correspond with 16.37 grains of ammonio-citrate in each fluid ounce of the tincture examined. The slight discrepency is probably due to some difference in graduated measures.

